# Determinants of cervical cancer screening utilization among HIV-positive women, in public general hospitals of Central Zone, Tigray, Ethiopia, 2020: Case-control study

**DOI:** 10.1371/journal.pone.0289042

**Published:** 2023-12-12

**Authors:** Tsega Gebremichael Gebremeskel, Merkeb Zeray Gebretatios

**Affiliations:** 1 Department ofEpidemiology and Biostatistics, College of Health Sciences, Aksum University, Aksum, Ethiopia; 2 Department of General Public Health, College of Health Sciences, Aksum University, Aksum, Ethiopia; Addis Ababa University School of Public Health, ETHIOPIA

## Abstract

**Introduction:**

Cervical cancer is the second leading cause of cancer-related morbidity and mortality in Ethiopia. Evidence showes that, despite the magnitude and severity of cervical cancer, utilization of cervical cancer screening in the study area among HIV-positive women is low.

**Objective:**

This study aimed to assess the determinants of cervical cancer screening utilizationamong HIV-positive women in general public hospitals in the central zone of Tigray, Ethiopia.

**Method:**

This study was a facility-based unmatched case-control study conductedamong HIV-infected womenin public general hospitals in the central zone of Tigray. Cases were HIV infected women not screened for cervical cancer, and controls were HIV infected women screened for cervical cancer. A total of 800participants (400 cases and 400 controls) wereselected using systematic random sampling with a 1:1 case-to-control ratio.Data collection was done using a pre-tested interviewer-administered questionnaire and a medical record review.The determinants of cervical cancer screeningutilization were identified through binary logistic regression.

**Result:**

Significant determinants of cervical cancer screening utilization among HIV-infected women in the central zone of Tigray werebeing in the age group of 18–30 [AOR = 0.46, 95% CI = 0.22, 0.98], living in rural areas [AOR = 0.47, 95% CI = 0.26, 0.87], no formal education [AOR = 0.25, 95% CI = 0.14, 0.45] and primary education [AOR = 0.28, 95% CI = 0.16,0.49], not working outside the home [AOR = 0.21, 95% CI = 0.10, 0.44], poor knowledge about cervical cancer [AOR = 0.29, 95% CI = 0.19, 0.44], and about cervical cancer screening [AOR = 0.44, 95% CI = 0.28, 0.70], and unfavorable attitudes toward cervical cancer screening [AOR = 0.52, 95% CI = 0.34, 0.79].

**Conclusion:**

Being in the age group of 18–30, living in rural areas, lacking formal education or havingonly primary level education, not working outside of the home, having poor knowledge of cervical cancer and screening,and having unfavourable attitudestowards cervical cancer screeningare significant determinat factors in cervical cancer screening utilization among HIV-infected women in the central zone of Tigray, Ethiopia. Considering such factors during the design of policies and programs could enhance the utilization of cervical cancer screening in the region.

## Introduction

Cervical cancer is a malignancy primarly caused by the human papillomavirus (HPV) a sexually transmitted infection (STI) [1–3].

Cervical cancer is a major public health problem worldwide. In 2018, approximately 569,847 new cases of cervical cancer were diagnosed worldwide, and approximately 311,365 deaths were attributed to cervical cancer in that same year [4]. The majority of thesenew cases and deaths occurred in low- and middle-income countries. In sub-Saharan Africa,cervical cancer accountsfor 22.2% of all cancers in women. It isalso the leading cause of death from cancer among women in the region [5,6]. Sub-Saharan Africa has a double burden of humanpapilloma virus (HPV) and HIV infection. The presence of the HIV acquired immune deficiency syndrome (AIDS) epidemic in the region raises the burden of cervical cancer to a serious level [6,7]. In East Africa, age-adjusted cervical cancer incidence and mortality rate per 100,000 women is 40.1 and 30, respectively [4].

In Ethiopia, cervical cancer ranks as the second leading cause of female cancer, with an age- adjusted incidence rate of 18.9 and a mortality rate of 15.3 per 100,000 women [4]. The prevalence of HIV/AIDS among women aged 15–49 is 1.2% [8]. This indicates that around 378,000 women over age 15 living with HIV in Ethiopia are at risk of developing cervical cancer [4], as HIV infected women are four to five times more likely to acquire persistent HPV infection that can cause cervical abnormalities and cervical cancer, andalso have a reduced capacity to clear HPV-infection as compared to HIV-negative women [9].

Unlike other gynecological cancers,cervical cancer is potentially preventable. With effective screening programs,the incidence, morbidity, and mortality of cervical cancer can be reduced [1,3,10–12]. Moreover, regular screening of HIV-positive women followed by immediate treatment to detect precancerous lesions can potentially prevent 80% of cervical cancer cases [13].

Even though cervical cancer is a significant risk for HIV-positive women,cervical cancer screening utilization among HIV-positive women in the Tigray region is very low compared to the national recommended coverage of 80% [12].

Various factorscan affect cervical cancer screeningutilization [14–17]. Little is known, however, about the determinants of cervical cancer screening-utilization among HIV-positive women. Previous studies [17,18] conducted in the country among HIV-positive womenhave useda cross-sectional study design, and have reported that cervical cancer screening utilizationis low (7.8%). These studies did not explore the determinants of cervical cancer screening utilization among HIV-positive women. Understanding the determinants related to cervical cancer screening utilization among HIVinfected womenisanentry point to improving cervical cancer screening utilization.

Assessing such factors will provide important information for policymakers, planners, and implementersof cervical cancer prevention and control programs, so that intervention strategies can be designed to target key determinants of cervical cancer utilization.Accordingly, the main aim of this study is to identify the determinants of cervical cancer screening utilization among HIV-infected women using a case-control study design.

## Methods and materials

A facility-based unmatched case-control study was conducted among HIV-positive patients in public general hospitals inthe central zone of Tigray, northernEthiopia.The study was conducted in public general hospitals,where routine cervical cancer screening and ART services are available. The capital city of the zone is Aksum town, which is located 1024 kilometers north of Addis Ababa. There are 66 health facilities in total(1 referral hospital, 3 general hospitals, 6 primary hospitals, and 56 health centers) providing different health services in the zone. A cervical cancer screening service is only available at the two general hospitals in the zone. Currently, a total of 3168 people are on ART at the general hospitals. Out of them, 1826 are women aged 18 and above. The data collection period wasfromJanuary 29 to March 15, 2020.

The study was conducted among HIV-positive women whowere attending regular follow up appointments at the general hospital ART clinics, both those who had not been screened for cervical cancer and those that had been.The inclusion criteria were: HIV-positive women above 18 years old not screened for cervical cancer, and HIV-positive women above 18 years oldscreened for cervical cancer. HIV-positive women diagnosed with a psychiatric disorder of aggressive behavior, who were unable to concentrate, and who were diagnosed with a serious illness (critical illness) by physician were excluded from the study.

### Sample size determination

The sample size for this study was determined by double population proportion using Epi-Info version 7.0.8.3 with the assumption of a confidence interval of 95%, a power of 80%, and aratio of cases to controls of1:1. Taking the percent of controls exposed to 91.7 and the OR of 2.57 of ever giving birth [19], the final sample size was 800 (400cases and 400 controls).

### Sampling

A purposeful sampling technique was used to select two public hospitals from the general hospitals in the central zone,where routine cervical cancer screening and ART services are being provided.The proportional allocation of study participants to the hospitals was determined according to the average monthly ART clinic client flow, based on the review of patient visits recorded in registration books. The Study participants selected were HIV positive women aged 18 and above, who were in regular follow up appointment at the general hospital ART clinics for six months and above, attending follow up appointment at the general hospital ART clinics during data collection period (from January 29—March 15, 2020), and both those who had not been screened for cervical cancer and those that had been screened for cervical cancer at least once in their life time. Cases and controls were selected using systematic random sampling until the required sample size was reached. Both cases and controls were selected from the general hospitals. The samples were representatives of the larger population as all general hospitals that provides routine ART folloup and cervical cervical cancer screening were included in the study and the study participants were selected using probability sampling method.

### Study variables

**Dependent variable**:

**Cervical cancer screening utilization**:was categorized into cases and controls (cases = 0, controls = 1).

**Independent variables**:

**Socio-demographic factors**:

Age: (18–30 = 1, 31–40 = 2, 41 and above = 3)

Residence:(urban = 1 and rural = 2)

Religion: (orthodox = 1, and other = 2)

Marital status:(single = 1, married = 2, divorced = 3, and widowed = 4)

Educational status: (noformal education = 1, primary education = 2, and secondary and above = 3), and Occupation: (housewife = 1, self-employed = 2 and government employee = 3)

### Reproductive health-related factors

Age at first sex: (less than eighteen = 1, and morethan or equal to eighteen = 2)

Multiple sexual partners:(yes = 1, andno = 2)

Number of births: (one = 1, twoand three = 2, three, four and above = 3)

Ever give birth: (yes = 1, and no = 2)

History of contraceptive use: (yes = 1, and no = 2)

Family history of cervical cancer: (yes = 1, and no = 2) and

History of STIs: (yes = 1 and no = 2)

### HIV/AIDS-related factors

WHO clinical stage of HIV/AIDS: (one = 1, two = 2, and three and above = 3),

Duration of HIVinfection: (less than four = 1, four upto eight = 2, and nine and above = 3) Duration on ART: (less than four = 1, four upto eight = 2, and nine and above = 3),

CD4 count: (lessthan 500 = 1,and 500 and above = 2),

### Knowledge- related factors

Knowing a cervical cancer victim: (yes = 1, and no = 2),

Knowledge about cervical cancer, and screening: (good knowledge = 1, and poor knowledge = 2)

### Attitude-related factor

Attitude toward cervical cancer screening:(favorable attitude = 1, and unfavorable attitude = 2).

### Definition of terms

**Cases:** HIV-positive women not screened for cervical cancer,**Controls:** HIV-positive women who had had cervical cancer screeningat least once in theirlifetime.**Cervical cancer screening:** steps or procedures taken to identify HIV-positive women with any form of cervical change and those without any form of cervical change using visual inspection with acetic acid

**Knowledge:**The scores for each study participant was computed for knowledge about cervical cancer (based on seven questions)and for knowledgeabout cervical cancer screening(based on five questions). Next, the mean score was calculated. The mean score of knowledge about cervical cancer was **4.8.**Those whoscored less than **4.8**were considered to have **poor knowledge**about cervical cancer, and those who scored greater than or equal to **4.8**were considered to have**good knowledge**about cervical cancer.The mean score of knowledge toward cervical cancer screening was **2.9**. Those whoscored less than **2.9**were considered to have **poor knowledge**about cervical cancer screening, and those who scored greater than or equal to **2.9**were considered to have**good knowledge** about cervical cancer screening

**Attitude:** Similarly, the score was calculated for each study participant for attitude towards cervical cancer screening (based on three questions). The mean score was**2.1**. Those who scored **2.1** and abovewere considered to have a **Favorable attitude**towards cervical cancer screening, and those who scored below **2.1** considered to have an**unfavorable attitude** towardscervical cancer screening.

### Data collectiontools and techniques

Data wascollected using a pre-tested interviewer-administered questionnaire and a medical record review.The questionnaire has four sections; namely socio-demographic, HIV-related, reproductive, and individual health-related characteristics. The questionnaire was developed by reviewing relatedliteratures. The English version of the questionnaire was translatedinto Tigrigna by a person who is fluent in English, and Tigrigna was then retranslated back to the English version to check the appropriateness of the tool, consistency, language clarity, and accuracy by another person.The data collection tool was pre-tested on 10% of the sample population who were not included in the study group, in shire Town. Necessary modification was made to the questionnaire based on the pre-testing results.

The medical data record review was done to gather information such as WHO clinical staging and CD4 countfrom January 29 to March 15, 2020. Three qualified ART service providers from each hospitalwere recruited to collect the data, and three BSc nursessupervised the data collection process.Before the actual data collection, data collectors and supervisors were trained for two days by the principal investigator on interviewing techniques, objectives, methodologies, and tools of the study.

### Data analysis

The data wasentered into Epi-data software version 3.1 and exported to Stata version 13 for analysis.The Hosmer-Lemeshow goodness of fit test was used to check whether the model adequately fits the data in this study. The p-value was insignificant,showing that the data fit the model.Descriptive statistics were obtained to describe the socio-demographic, HIV/AIDS-related, individual, and reproductive health-related characteristics of the respondentsas well as to display the proportion of different categories of each predictor variable with cases and controls. The mean with SD wasused to summarize continuous non-normally distributed variables. The mean was used to categorize the scores for knowledge and attitude.

A bivariate logistic regression analysis was done to assess the presence of an association between the dependent and independent variables. These variables, whose bivariate analysis has a p-value of 0.2 or less,were candidates for the multivariable analysis. The result was presented using a p-value. Multivariable logistic regression analysis was done with all the candidate variables found in the bivariate analysis to obtain cofounder adjusted estimatesand was reported using an adjusted odds ratio (AOR) with a 95% confidence interval (CI). AP-value of less than 0.05 was used to identify factors significantly associated with cervical cancer screening utilization. Multicollinearity among determinant variables was checked using the variance inflation factor (VIF), and a VIF value of less than 10 was declared as an absence of ahigh degree of multicollinearity between determinant variables.Six variables with a high VIF (>10) were excluded from the final model due to the high degree of multicollinearity.

### Ethical consideration

Ethical approvalwas obtained from the ethical review board of the Aksum University College of Health Science. A letter of support was written tothe Tigray regional health bureau (TRHB) from Aksum University College of Health Science to obtain an official permission letter.Written informed consent was obtained from the participants. All information that was obtained from the women was kept confidential.

## Result

A total of 800 study participants (400 cases and 400 controls)participated in the study. The response rate was 100%.

The mean age was for cases 35.61 with SD (±5.85) and for controls 37.57 with SD (±5.02). 274 (68.50%) of the cases and 369 (92.25%) of the controls liveds in urban areas. For marital status 223 (55.75%) of the cases and 160 (40.00%) of the controls were married. The majority of the cases 379 (94.75%) and controls 361 (90.75%) were Orthodox Christian followers. Regardingeducational status,half (50.50%) of the cases had no formal education; However, 59.75% of the controls attended secondary school or above. Furthermore, more than half of the cases (243, 60.75%) and controls (235, 58.75%) were housewive and self-employed,respectively. (**Table 1**).

**Table 1:** Socio-demographic characteristics of study participants in the central zone of Tigray, northern Ethiopia, 2020.

Variables	Cases n (%) n = 400	Controls n (%) n = 400	Total n (%)
**Age**			
18–30	89(22.25)	46(11.50)	135(16.88)
31–40	240(60.00)	254(63.50)	494(61.75)
41 and above	71(17.75)	100(25.00)	171(21.38)
**Residence**			
Urban	274(68.50)	369(92.25)	643(80.38)
Rural	126(31.50)	31(7.75)	157(19.63)
**Marital status**			
Single	19(4.75)	14(3.50)	33(4.13)
Married	223(55.75)	160(40.00)	383(47.88)
Divorced	47(11.75)	72(18.00)	119(14.88)
Widowed	111(27.75)	154(38.50)	265(33.13)
**Religion**			
Orthodox	379(94.75)	361(90.25)	740(92.50)
Other	21(5.25)	39(9.75)	60(7.50)
**Educational status**			
No formal education	202(50.50)	66(16.50)	268(33.50)
Primary	146(36.50)	95(23.75)	241(30.13)
Secondary and above	52(13.00)	239(59.75)	291(36.38)
**Occupation**			
Housewife	243(60.75)	51(12.75)	294(36.75)
Self-employed	134(33.50)	235(58.75)	369(46.13)
Government employee	23(5.75)	114(28.50)	137(17.13)

### HIV/AIDS related factors of the study participants

Most of the cases (338, 84.50%) and controls (376, 94.00%), were in WHO clinical stage one. 162(40.60%) of the cases and 236 (60.36%) of the controls werediagnosed as HIV positive at minimum nine years ago. However, about 145 (36.34%) of the cases started ART betweenfour to eight years agoand half of the controls (209, 53.45%) started ART a minimum of nine yearsago. As a result, half of the cases (202, 50.50%) and more than half of the controls (228, 57.00%) had a CD4 count less than 500cells/μland greater than or equal to 500 cells/μl respectively (**Table 2**).

**Table 2:** HIV AIDS related factors of respondents inthe central zone of Tigray, northern Ethiopia, 2020.

Variables	Cases n (%) n = 400	Controls n (%) n = 400	Total n (%)
**WHO clinical stage of HIV AIDS**			
1	338(84.50)	376(94.00)	714(89.25)
2	51(12.75)	15(3.75)	66(8.25)
3 & 4	11(2.75)	9(2.25)	20(2.50)
**Duration of HIV infection**			
<4 years	106(26.57)	48(12.28)	154(19.49)
4–8 years	131(32.83)	107(27.37)	238(30.13)
9 & above	162(40.60)	236(60.36)	398(50.38)
**Duration on ART**			
<4 years	114(28.57)	50(12.79)	164(20.76)
4–8 years	145(36.34)	132(33.76)	277(35.06)
9 & above	140(35.09)	209(53.45)	349(44.18)
**CD4 count of the patient**			
**<**500 μl	202(50.50)	172(43.00)	374(46.75)
≥500 μl	198(49.50)	228(57.00)	426(53.25)

### Reproductive and individual health related characteristics of respondent

The mean and standard deviation of age at first sexual intercourse of cases and controls were 17.51±2.50 and 17.60±3.153 years, respectively.The majorityof the cases (358, 89.50%) and controls (358, 89.50%) had given birth. In addition, nearly all of the cases (398, 99.50%)and controls (391, 97.75%) had no family history of cervical cancer. Similarly,among the study subjects, 384 (96.00%) of the cases and 378 (94.50%) of the controls did not know anyone with cervical cancer.

225 (56.25) of the cases and 245 (61.25%) of the controls had a history of multiple sexual partners; 264 (66.00%) of the cases and 208 (52.00%) of the controls had a history of contraceptive use. Furthermore, among the respondents, 209 (52.25%) of the cases had poor knowledge of cervical cancer screening, and 333 (83.25%) of the controls had good knowledge about cervical cancer screening. Lastly, 278 (69.50%) of the cases had an unfavorable attitude toward cervical cancer screening, and 253 (63.25%) of the controls had a favorable attitude toward cervical cancer screening (**Table 3**).

**Table 3:** Reproductive and individual health related factors of respondentsin the central zone of Tigray, northern Ethiopia, 2020.

Variables	Cases n (%) n = 400	Controls n (%) n = 400	Total n (%)
**Age at first sex**			
<18 years	200(50.00)	202(50.50)	402(50.25)
≥18 years	200(50.00)	198(49.50)	398(49.75)
**Ever give birth**			
Yes	358(89.50)	358(89.50)	716(89.50)
No	42(10.50)	42(10.50)	84(10.50)
**Number of birth**			
One	73(20.39)	84(23.46)	157(21.93)
Two & three	236(65.92)	208(58.10)	444(62.01)
Four & above	49(13.69)	66(18.44)	115(16.06)
**Family history of cervical cancer**			
Yes	2(0.50)	9(2.25)	11(1.38)
No	398(99.50)	391(97.75)	789(98.62)
**Know someone with cervical cancer**			
Yes	16(4.00)	22(5.50)	38(4.75)
No	384(96.00)	378(94.50)	762(95.25)
**History of multiple sexual partner**			
Yes	225(56.25)	245(61.25)	470(58.76)
No	175(43.75)	155(38.75)	330(41.26)
**History of STI**			
Yes	142(35.50)	159(39.75)	301(37.63)
No	258(64.50)	241(60.25)	499(62.38)
**History of contraceptive use**			
Yes	264(66.00)	208(52.00)	472(59.00)
No	136(34.00)	192(48.00)	328(41.00)
**Knowledge about cervical cancer**			
Good knowledge	139(34.75)	299(74.75)	438(54.75)
Poor knowledge	261(65.25)	101(25.25)	362(45.25)
**Knowledge about cervical cancer screening**			
Good knowledge	191(47.75)	333(83.25)	524(65.50)
Poor knowledge	209(52.25)	67(16.75)	276(34.50)
**Attitude on cervical cancer screening**			
Favorable attitude	122(30.50)	253(63.25)	375(46.87)
Unfavorable attitude	278(69.50)	147(36.75)	345(53.13)

### Determinant factors of cervical cancer screening utilization

Binary logistic regression was done to identify the determinants of cervical cancer screening utilization. From the bivariate analysis,the following variables were selected for the multivariable analysis at a cutoff p-value of 0.2 or less: WHO clinical stage of a patient, CD4 count of a patient, age of mother, residence, marital status of mother, religion, educational status, mother employment, number of births, duration of HIV infection, duration on ART, family history of cervical cancer, multiple sexual partners, history of contraceptive use, knowledge about cervical cancer and screening, and attitude toward cervical cancer screening.

In the multivariable analysis, the following variables were independent determinants of cervical cancer screening utilization: from the socio-demographic factors,the age of the mother, residence, educational status, and employment status;from the individual health-related factors,knowledge about cervical cancer and cervical cancer screening and attitude toward cervical cancer screening.

Keeping other predictors constant, those between the ages of 18 and 30 had a 50%[AOR = 0.46, 95% CI = 0.22,0.98] reduced level ofutilizing cervical cancer screening compared to those who were in the age group of 41 and above. The odds of utilizing cervical cancer screening among womenliving in rural areas were53% lower [AOR = 0.47, 95% CI = 0.26, 0.87] comared to womenliving in urban areas.

Mothers who did not attend formal education and mothers who attended only prmary education were 75% and 72% less likely to utilize cervical cancer screening than those who attended secondary school and above [AOR = 0.25, 95% CI = 0.14, 0.45 and AOR = 0.28, 95% CI = 0.16, 0.49] respectively.Not working out side the house decreased the probability of cervical cancer screening utilization by 79% [AOR = 0.21, 95% CI = 0.10, 0.44] compared to women working outside the home as government employee.

The likelihood of utilizing cervical cancer screening among women who had poor knowledge about cervical cancer was71% lower [AOR = 0.29, 95% CI = 0.19, 0.44] as compared towomen with good knowledge about cervical cancer. Women with poor knowledge about cervical cancer screening hada 56% [AOR = 0.44, 95% CI = 0.28, 0.70] lower utilization of cervical cancer screeningcompared towomen who had good knowledge about cervical cancer screening.

Women with an unfavorable attitude towards cervical cancer screening were48% [AOR = 0.52, 95% CI = 0.34, 0.79] less likely to access cervical cancer screening compared to women who had a favorable attitude toward cervical cancer screening (**Table 4**).

**Table 4:** Multivariable analysis of factors associated with cervical cancer screeningutilization among respondents inthe central zone of Tigray, northern Ethiopia, 2020.

Variables	Cases n (%)	Controls n (%)	AOR(CI)
**Age**			
18–30	89(22.25)	46(11.50)	0.47 (0.22, 0.98)*
31–40	240(60.00)	254(63.50)	0.75 (0.45, 1.26)
41 and above	71(17.75)	100(25.00)	1
**Residence**			
Urban	274(68.50)	369(92.25)	1
Rural	126(31.50)	31(7.75)	0.47(0.26, 0.87)*
**Educational status**			
No education	202(50.50)	66(16.50)	0.25(0.13, 0.45)***
Primary	146(36.50)	95(23.75)	0.30(0.16, 0.49)***
Secondary and above	43(10.75)	190(47.50)	1
**Occupation**			
Housewife	243(60.75)	51(12.75)	0.21(0.10, 0.44)***
Self-employed	134(33.50)	235(58.75)	1.14(0.59, 2.22)
Government employee	23(5.75)	114(28.50)	1
**CD4 count of the patient**			
**<**500 μl	202(50.50)	172(43.00)	0.80(0.53,1.22)
≥500 μl	198(49.50)	228(57.00)	1
**Number of birth**			
One	73(20.39)	84(23.46)	0.66(0.33, 1.34)
Two & three	236(65.92)	208(58.10)	0.67(0.38, 1.18)
Four & above	49(13.69)	66(18.44)	1
**History of multiple sexual partner**			
Yes	225(56.25)	245(61.25)	0.88(0.58, 1.34)
No	175(43.75)	155(38.75)	1
**History of contraceptive use**			
Yes	264(66.00)	208(52.00)	1.51(0.97, 2.33)
No	136(34.00)	192(48.00)	1
**Knowledge about cervical cancer**			
Good knowledge	139(34.75)	299(74.75)	1
Poor knowledge	261(65.25)	101(25.25)	0.29(0.19, 0.44)***
**Knowledge about cervical cancer screening**			
Good knowledge	191(47.75)	333(83.25)	1
Poor knowledge	209(52.25)	67(16.75)	0.44(0.28, 0.70)***
**Attitude on cervical cancer screening**			
Favorable attitude	122(30.50)	253(63.25)	1
Unfavorable attitude	278(69.50)	147(36.75)	0.52(0.34, 0.79)**

Key: AOR = adjusted odds ratio, CI = confidence interval, 1 = reference category

* P value<0.05

** p value<0.01

*** p value<0.001.

**NB:** Marital status, religion,WHO clinical stage of HIV AIDS, Duration of HIV infection, Duration on ART, and Family history of cervical cancer was excluded from the final model due to the high degree of multicollinearity.

## Discussion

Thisstudy aimed to identify the determinants of cervical cancer screening utilization among HIV-infected women in public general hospitals inthe central zone of Tigray. Accordingly, women in the age groupof 18–30, living in rural areas, with no formal education or only primary education, not working outside the home, poor knowledge about cervical cancer and screening, and unfavorable attitudes towards cervical cancer screening were found to bedeterminants of cervical cancer screening utilization.

This study showed lower cervical cancer screening utilization among HIV-positive women in the 18–30 age group as compared to as compared to women aged 41 and above. This result is in line with previous studies donein Tigray,Ethiopia [19], Dessie Town, North East Ethiopia [20], and Addis Ababa, Ethiopia [21], which revealed that women in the 18–29 age cohort were less likely to access cervical cancer screeningas compared to women in the (40–49) age cohort. A possible explanation may be that younger womendo not have adequate exposure to informationabout cervical cancerscreening, whereas older womenmay have more exposure to health information when visiting health facilities for different services. Additionally,Ethiopian health policy advises cervical cancer screening for women over the age of 30 [20], which may lead younger women to view themselves as lower risk groups forcervical cancer related morbidity and mortality.

Results of this study revealed that HIV-infected women who live in rural areas had a lowerlikelihood of utilizing cervical cancer screening than women living in urban areas. This finding is similarto studies done in Aksum, Tigray, Ethiopia [22] and Addis Ababa, Ethiopia [21] which showed that women living in rural areas were less likely to access cervical cancer screening than women living in urban areas.This may be due in part to the fact thatthe majority of the screening facilities in both Tigray specifically and Ethiopia generally are located in urban areas [21].

This study demonstrated that HIV-positive women with no formal education or only primary education had a lowerprobability ofcervical cancer screening utilization than women with secondary educationand above. This finding is consistent with studies conducted in Aksum, Tigray, Ethiopia [22] and Shabadino District, southern Ethiopia [23]. A possible explanation for this finding is thateducation can increase women’s accessto information from different sources, and educated women may have better knowledge and understanding of thebenefits of cervical cancer screening service utilization.

This study also showed that women who did not work outside the home wereless likely to utilize cervical cancer screening than women working for the government. This finding is in line with studies conducted in Addis Ababa, Ethiopia [17] and Dessie Town, North East Ethiopia [20], which showed that the probability of cervical cancer screening was higher among government employees than among those who were non-employed. A possible explanation for these results is that women who do not work outside the home may not have exposure to information about cervical cancer and its screening from different sources, such as workplaces and work colleagues [20].

Knowledge status was significantly associated with cervical cancer screening utilization. HIV-infected women with poor knowledge about cervical cancer and its screening had lower odds of cervical cancer screening utilization than HIV-infected women with good knowledge of cervical cancer and its screening. This result is similar to a finding obtained from a study conducted in Almata, Tigray, Ethiopia [18]and a study conducted in Tigray, Ethiopia [19], Shabadino District,Southern Ethiopia [23], Addis Abeba, Ethiopia [21], and northern Malaysia [24], which showed that lack of knowledge of cervical cancer and screening were barriers to utilizing cervical cancer screening. It seems evident that not having adequate knowledge of cervical cancer and screening could lead to poor utilization of the service.

This study also showed that HIV-positive women who had an unfavorable attitude toward cervical cancer screening hada lower likelihood ofutilizing cervical cancer screening compared toHIV-positive women with a favorable attitude toward cervical cancer screening. This finding was demonstrated by astudy done in Tigray, Ethiopia [19], Dessie Town, North East Ethiopia [20] and northern Malaysia [24]. Because action may be linked to pre-existing attitudes, a woman with an unfavourable attitude about cervical cancer screening may be less likely to utilize the cervical cancer screening.

This study did have a limitiation. Namely, it did not consider the regularity of the cervical cancer screening among HIV-positive women.

## Conclusion

This study identifiedage, residential area, educational status, employment status, knowledge, and attitude towards cervical cancer screening as determinant factorsof cervical cancer screening utilization among HIV infected women in public general hospitals inthe central zone of Tigray in 2020.

Based on the findings, the study makes the following recommendations:

Desiging tools that will increase cervical cancer screening utilization in women in the age group of 18–30

Ensuring cervical cancer screening in proximity to where women are or women have the means to reach to cervical cancer screening places.

Designing of tools that will have an impact on the cervical cancer screening utilization of women with no/primary level of education.

Integratingcervical cancer screening awareness into regualr heatlh education and promotion activities.

## Supporting information

S1 File Data set. (DTA)

S2 File Questionnaire English version. (DOCX)

S3 File Questionnaire in local language. (DOCX)

## Abbreviations

ART: Anti-Retroviral Treatment
HIV/AIDS: Human Immune Deficiency Virus/ Acquired Immune Deficiency Syndrome
HPV: Human Papilloma Virus; STI- Sexually Transmitted Infection
VIF-: Variance Inflation Facto
WHO: World Health Organization
