# Supplementary material for: Determinants of cervical cancer screening utilization among HIV-positive women, in public general hospitals of Central Zone, Tigray, Ethiopia, 2020: Case-control study
Source: PLoS One. 2023 Dec 12;18(12):e0289042. doi: 10.1371/journal.pone.0289042 (PMC10715646; doi:10.1371/journal.pone.0289042)
Supplement: S2 File — (DOCX) [file pone.0289042.s002.docx]

# Questionnaire English version

**Aksum University**

**School of Public Health**

**Structured Questionnaire English Version**

**Determinants of cervical cancer screening utilization among HIV infected women**
**in public general hospitals of central zone of Tigray, North Ethiopia. Case control Study.**

| General information | | | |  | |
| --- | --- | --- | --- | --- | --- |
| Questions | | Response & Coding Categories | | Skip | |
| Date of data collection | | dd/mm/yy | |  | |
| Code of data collectors | |  | |  | |
| Code of the facility | |  | |  | |
| Code of the questionnaire | |  | |  | |
| Total ART client in the facility | |  | |  | |
| Patient ID WHO | |  | |  | |
| WHO clinical disease stage of the patient | |  | |  | |
| Patient CD4 count | |  | |  | |
| Section I Socio-demographic Characteristics | | | | | |
| Name of the hospital | | | | | |
| SN | Questions | | Response & Coding Categories | | Skip |
| 101 | How old are you?(Complete in years) | | --------------------------- | |  |
| 102 | What was your age at first sex | |  | |  |
| 103 | Where do you live | | 1.urban  2.rural | |  |
| 104 | What is your marital status | | 1 Single  2. Married  3. Divorced  4. Widowed | |  |
| 105 | What is your religion | | 1Orthodox 2.Muslim  3. Protestant  4 catholic  5. Other Specify…………. | |  |
| 106 | What is your educational Status | | 1. No education 2. Primary 3. Secondary 4. college and above | |  |
| 107 | What is your work? | | 1. Housewife 2. Self-employees 3. Government employees 4. .Others, specify____ | |  |
| 108 | Have you ever give birth? | | 1. Yes  2. No | | If NO skip to Q.111 |
| 109 | How many births have you given? | | 1.One  2. Two  3.Three  4.four and above | |  |
| 110 | When was your HIV diagnosis? | | ……………….(year or duration) | |  |
| 111 | When was you started follow up in ART care……? | | …………………(year or duration) | |  |
| 112 | Family history of cervical cancer | | 1. Yes  2. No | |  |
| 113 | Do you know a person with a cervical cancer | | 1. Yes 2. No | |  |
| 114 | Have you ever had multiple sexual partners? | | 1.YES  2.NO | |  |
| 115 | Did you use contraceptive | | 1. Yes  2. No | | If no go to  Q. 200 |
| Section II Knowledge of the study participants on cervical cancer and cervical cancer screening | | | | |  |
| SN | Questions | | Response & Coding Categories | | Skip |
| 200 | Have you ever heard about cervical cancer? | | 1.Yes  2. No | | If No Go to Q.210 |
| 201 | From where did you hear about cervical cancer for the last time? | | 1. Media (Television, Radio, Magazine, Brochures)  2. Health professional  3. School  4. Family  5. Friends  6. Other, specify………. | |  |
| 202 | What are the symptoms of cervical cancer? | | 1. Vaginal bleeding  2. Foul vaginal discharge  3. Pelvic or back pain  4. Post coital bleeding  5. I do not know  6. Other, specify………………. | |  |
| 203 | Do you know factors that causes cervical cancer | | 1. Yes  2. No | | If NO skip to Qu 205 |
| 204 | What are the risk factors to cervical cancer? | | 1. Age  2. Early onset of sexual intercourse  3. STI infection  4. OCP use  5. Having multiple sexual partners 6. Family history of cervical cancer  7 Cigarette smoking  8. I do not know  9. Other, specify………………. | |  |
| 205 | Is cancer of the cervix can be transmitted from one person to another? | | 1. Yes 2. No 3. I don’t know | | If NO go to Q.207 |
| 206 | If yes, above, how is it transmitted? | | 1. Sexually transmitted [ ]  2. Through contact with the sick  3. Through air  4. Don’t know  5. Other specify……….. | |  |
| 207 | Is cervical cancer preventable disease? | | 1.Yes  2. No  3 I do not know | | If NO go to Q.210 |
| 208 | How can we prevent cervical cancer? | | 1. Avoid multiple sexual partners 2. 2. Avoid early onset sexual intercourse  3. Quit smoking  4. Through vaccination  5. Through screening services  6. Other, specify……………. | |  |
| 209 | Is cervical cancer curable (treatable)? | | 1. Yes  2. No  3. I do not know | | If NO go to  Q.212 |
| 210 | Have you ever heard about cervical cancer screening? | | 1. Yes  2. No  3. I do not know | | If no skip to Q.301 |
| 211 | If yes to Q. 210 From where did you hear about cervical cancer screening for the last time? | | 1. Media (Television, Radio, Magazine, Brochures)  2. Health professional  3. School  4. Family  5. Friends  6. Other, specify………. | |  |
| 212 | What is the aim of cervical cancer screening? | | 1. To prevent cervical cancer  2. To early detection of cervical cancer  3. To treat cervical cancer  4. Other, specify | |  |
| 213 | When HIV positive woman should have screening? | | 1. When menstruation starts  2. As soon as sexually active  3. At the age of 30  4. When start having children  5. After menopause  6. I do not know  7. Other, specify …………… | |  |
| 214 | How frequent, cervical cancer screening should be done for HIV positive women? | | 1. Once every year  2. Once every two year  3. Once every three years  4. Once every 5 years  5. I do not know  6. Others specify……… | |  |
| 215 | Do you know any health institution which provides cervical screening  in your area | | 1.Yes  2 No | |  |
| Section I Attitude for cervical cancer and screening | | | | | |
| 301 | Do you see yourself as being at risk of cervical cancer? | | 1. Yes 2. No | |  |
| 302 | One can have cervical cancer through unsafe sexual practice | | 1. Yes 2. No | |  |
| 303 | One cannot be cured from cervical cancer once a diagnosis is made. | | 1. Yes 2. No | |  |
| 304 | Cervical cancer is more common to women who are HIV positive | | 1. Yes 2. No | |  |
| 305 | Do you think that you can have cervical cancer but not symptoms | | 1. Yes 2. No | |  |
| 306 | Via screening prevent from cervical cancer | | 1. Yes 2. No | |  |
| 307 | Are you willing to regularly consult a health care provider for screening of cervical cancer? | | 1. Yes 2. No | |  |
| 308 | In your opinion, who should be screened? | | 1. All women of child bearing age 2. Only women with symptoms suggestive of cancerous cervix  3. HIV positive women  4. Don’t know | |  |

Thank you
