# Supplementary material for: Determinants of cervical cancer screening utilization among HIV-positive women, in public general hospitals of Central Zone, Tigray, Ethiopia, 2020: Case-control study
Source: PLoS One. 2023 Dec 12;18(12):e0289042. doi: 10.1371/journal.pone.0289042 (PMC10715646; doi:10.1371/journal.pone.0289042)
Supplement: S3 File — (DOCX) [file pone.0289042.s003.docx]

# Questionnaire in local language

**ናይ ስምምዕ ቅፅ**

ጥዕና ይሃበለይ!………………………………………እበሃል፡፡ስረሐይ አብ ኣክሱም ዩኒቨርሲቲ ጥዕና ሳይንስ ኮሌጅ ናይ ሕብረተሰብ ጥዕና ትምህርቲ ክፍሊ መምህር ፅንዓታዊ ፅሑፍ/ምርምር/ አብ ምክያድ ካብ ዝርከቡ፣መ/ር ፀጋ ገ/ሚካኤል ምስ ዝበሃላ ፅንዓት መካየዲት ክኸውን ከለኹ፤አብዚ ሆስፒታል ናይ ፀረ- ኤች አይ ቪ መድሓኒት ክትትል ንምውሳድ ዝመፃ አዴታት ብዛዕባ ናይ ኣፍ ደገ ማህፀን ካንሰር ቅድመ ምርመራ አብ ዝግባእ እዋን ንከይገብራ ዕንቅፋት ዝኾንወን ምክንያታት ንምፍላጥቃለ-መሕተት እገብር አለኹ፡፡ንስኽን እውን ናይቲ መፅናዕቲ ተሳታፊት ክትኮና ተዓዲምክን አለኽን፡፡

**ርእሲ ናይቲ መፅናዕቲ**፡አብ ማእኸላይ ዞባ ዝርከባ ኣብ ደመን ኤች አይ ቪ ቫይረስ ዘለወን ኣዴታት ናይ ኣፍ ደገ ማህፀን ካንሰር ቅድመ ምርመራ አብ ዝግባእ እዋን ንከይገብራ ዕንቅፋት ዝኾንወን ምክንያታት ንምፍላጥ ዝግበር መፅናዕቲ እዩ፡፡

**ዕላማ እቲ መፅናዕቲ:** ኣብ ደመን ኤች አይ ቪ ቫይረስ ዘለወን ኣዴታት ናይ ኣፍ ደገ ማህፀን ካንሰር ቅድመ ምርመራ አብ ዝግባእ እዋን ንከይገብራ ዕንቅፋት ዝኾንወን ምክንያታት ንምፍላጥ ዝግበር መፅናዕቲ ኮይኑ አብ ምምሕያሽ አጠቃቅማ ኣፍ ደገ ማህፀን ካንሰር ቅድመ ምርመራ ኣብ ደመን ኤች አይ ቪ ቫይረስ ዘለወን ኣዴታት አስተዋፅኦ ምግባር እዩ፡፡

**አሰራረሓ፡**-እዚ ኣብ ላዕሊዝተጠቐሰ መፅናዕቲ ንምክያድ ዝተፈላለዩ ሕቶታት ኣለዉ፡፡እዚ መፅናዕቲ ውፅኢታዊ ክኸውን ዝክእል ንስኽን ብእትህበኦ ትክክለኛ መልሲ ስለዝኾነ ነቶም ሕቶታት ብጥንቃቄ ንክትምልሳለይ ብትሕትና እሓትት፡፡ክብራህረሀልክን ትደልዮኦ ሕቶ እንተሃልዩ ከዓ ምሕታት ይከኣል እዩ፡፡

**ናይቲ መፅናዕቲ ጥቅምን ጉድኣትን**፡ኣብዚ መፅናዕቲ ብምስታፍክን ብቀጥታጥቕሚ ዘይከትረኽባ ትኽእላ ኢኽን፣ነገር ግን ናትክን ተሳትፎ ኣብ ዕላማ እቲ መፅናዕቲ፣ኣብ ደመን ኤች አይ ቪ ቫይረስ ዘለወን ኣዴታት ናይ ኣፍ ደገ ማህፀን ካንሰር ቅድመ ምርመራ ተሎ ንከይጅምራ ዝገብርወን ምክንያታትን፣ኣብ ዙርያ እዚ ዘለዉ ክፍተታት ምንጋር፣ትክክለኛ መፍትሒ ምሕባር ብጣዕሚ ኣድላዪ እዩ፡፡ኣብዚ መፅናዕቲ ብምስታፍክን ኣብ ባዕልኽን ይኹን ኣብ ስድራኽን ዝፍጠር ምንም ዓይነት ተፅዕኖ ሕዚ ይኹን ድሕሪ ሕዚ ኣይህሉን፡፡**ምስጢራውነት፡**-መጠየቕ ምሕዝነታውን ምሽጥራውን እዩ፡፡እትህባና ሓበሬታ ኩሉ ብቁፅሪ /ኮድ/ ዝቕመጥ ኮይኑ ስምክን ንኻሊእ ሳልሳይ ወገን ኣሕሊፍካ ኣይወሃብን፡፡ኩሉ ዝሃብክናና መልስን ሪኢቶን ንኻልእ ሰብ ኣሕሊፍካ ኣይወሃብን፡፡ዝኾነ ፀብፃብ እንትቀርብ ይኹን እንትሕተም ጥራሕ ሓበሬታኽን እዩ ዝቀርብ፡፡**ተሳትፎ**፡- እዚ ቃለ-መሕተት ኣብ ድሌት ዝተመስረተ እዩ፡፡ኣብዚ ቃለ-መሕተት ክትሳተፋ /ዘይክትሳተፋ ትኽእላ ኢኽን፡፡ሕቶታት ብከፊል ኮነ ብሙልእ ናይ ዘይምምላስ መሰል ኣለክን፡፡በዚ ኣብላዕሊ ዝተገለፀ ሓበሬታ መሰረት ሕዚ ኣብዚ ፅንዓት እዚ ንምስታፍ ፍቃደኛ እንተኮይንክን ክንቅፅል ፍቃደኛ እንተዘይኮይንክን ከዓ ኣብዚ ክነእክል?

**ናይ ተሳታፊት መሰማምዒ ውዕል**፡- ኣነ ኣብ ታሕቲ ፊርማይ ዘቀመጥኩ ግለሰብ ናይዚ መፅናዕቲ ዕላማ ተብራህሪሁለይ እዩ፡፡በቲ ዝተገለፀለይ ሓበሬታ መሰረት ኣብቲ መፅናዕቲ ክሳተፍ ተስማዕሚዐ ኣለኩ፡፡

ፌርማተሳታፊ--------------- ዕለት------------ ንምትሕብባረን ኣዚና ነመስግን፡፡

ቃለ-መሕተት ዘካየደ ሰብ ስም----------------------------ፌርማ---------ዕለት---------

ዘረጋገፀ ሱፐርቫይዘር ስም ------------------------------ፌርማ ------------ዕለት-------

**ቃለ-መሕተት**

| ጠቅላላ ሓበሬታ | | | | | | |  | |
| --- | --- | --- | --- | --- | --- | --- | --- | --- |
| ሕቶታት | | | | | | መልስንመማረፅታትን | Skip | |
| ዳታ ዝተሰብሰበሉ መዓልቲ | | | | | | መዓልቲ /ወርሒ/ዓ.ም |  | |
| ናይ ዳታ ሰብሳቢ ኮድ | | | | | |  |  | |
| ናይ ቃለመሕተት ኮድ | | | | | |  |  | |
| ጠቅላላ ናይ ፀረ-ኤችአይቪ መድሓኒት ተጠቀምቲ ናይቲ ትካል | | | | | |  |  | |
| ብውድብ ጥዕና ዓለም ናይ ኤች ኣይ ቪ ኤድስ ሕማም ደረጃ | | | | | |  |  | |
| ናይ ሕሙም CD4 ቁፅሪ | | | | | |  |  | |
| ክፍሊሓደ፡-ማሕበራዊን ኢኮነምያዊን ኩነታት | | | | | | | | |
| ናይ ሆስፒታል ስም | | | | | | | | |
| ተራ ቁፅሪ | ሕቶታት | | | | መልስን መማረፅታትን | | | Skip |
| 101 | ዕድመ ( ብዓመት) | | | | --------------------------- | | |  |
| 102 | ናይ መንበሪ ቦታኺ አበይ እዩ? | | | | 1.ከተማ  2.ገጠር | | |  |
| 103 | ናይ ሓዳር ኩነታትኪ እንታይ ይመስል? | | | | 1 ዘይተመርዐወት  2. ዝተመርዐወት  3.ዝተፋትሐት  4. ሰብኣይ ዘየብላ | | |  |
| 104 | ሃይማኖትኪ እንታይ እዩ? | | | | 1ኦርቶዶክስ  2.ሙስሊም  3.ፕሮቴስታንት  4 ካቶሊክ  5. ካልእ እንተኮይኑ ጥቀሲ | | |  |
| 105 | ደረጃ ትምህርትኪ ክንደይ እዩ? | | | | 1.መደበኛ ትምህርቲ ዘይብላ  2. ቀዳማይ ደርጃ  3. ካልአይ ደረጃ  4. ኮሌጅን ካብ ኮሌጅ ንላዕሊን | | |  |
| 106 | ስራሕኪ እንታይ እዩ? | | | | 1. ስራሕ ዘየብላ 2. ናይ ግሊ ስራሕ 3. ናይ መንግስቲ ስራሕ 4. ካልእ እንተኮይኑ ጥቀሲ | | |  |
| 107 | ናይ ቤተ-ሰብ ወርሓዊ አታዊ ክንደይ እዩ? | | | | …………..ብቅርሺ | | |  |
| 108 | ናይ በዓል ገዛኺ ደረጃ ትምህርቲ ክንደይ እዩ? | | | | 1. መደበኛ ትምህርቲ ዘየብሉ 2. ቀዳማይ ደርጃ 3. ካልአይ ደረጃ 4. ኮሌጅን ካብ ኮሌጅ ንላዕሊን | | |  |
| 109 | ናይ በዓል ገዛኺ ስራሕ እንታይ እዩ? | | | | 1.ናይ መንግስቲ ስራሕ  2. ናይ ግሊስራሕ  3.ስራሕ ዘየብሉ  4. ካልእ እንተኮይኑ ጥቀሲ | | |  |
| 110 | ወሊድኪ ዶ ትፈልጢ? | | | | 1. እወ  2. ኣይወለድኩን  መልስኪ ኣይወለድኩን እንተኮይኑ ናብ  ሕቶ ቁፅሪ 112 ሕለፊ | | | If NO skip  to Q.111  ኣይፈልጥን  እንተኮይኑ |
| 111 | ክንደይ ግዜ ወሊድኪ? | | | | 1.ሓደ  2. ክልተ  3.ሰለስተ  4.አርባዕተን ልዕሊኡን | | |  |
| 112 | ኣብ ደምኪ ኤች አይ ቪ ምህላዉ ንመጀመርያ ግዜ መዓዝ ፈሊጥኪ? | | | | ብዓመት | | |  |
| 113 | ናይ ኤች አይ ቪ መድሓኒት ክትትል መዓዝ ጀሚርኪ? | | | | ብዓመት | | |  |
| 114 | ኣብ ቤተ ሰብኩም ናይ ኣፍ ደገ ማህፀን ካንሰር ዘሎዎ ኣሎ ዶ ወይ ነይሩ ዶ? | | | | 1. እወ  2. የለን | | |  |
| 115 | ናይ ኣፍ ደገ ማህፀን ካንሰር ዘሎዎ ሰብ ትፈልጢ ዶ? | | | | 1. እወ 2. ኣይፈልጥን | | |  |
| 116 | | በዓል ገዛኺ ብዛዕባ ናይ ኣፍ ደገ ማህፀን ካንሰር ይፈልጥ ዶ? | | | 1. እወ 2. ኣይፈልጥን 3. ኣይፈለጥኩን | | |  |
| 117 | መከላከሊ ጥንሲ ትጥቀሚ ዶ? | | | | 1. እወ  2. ኣይጥቀምን  መልስኪ ኣይጥቀምን እንተኮይኑ ናብ ሕቶ  ቁፅሪ 200 ሕለፊ | | | If no go to  Q 118 |
| 118 | ንሕቶ ቁፅሪ 117 መልስኪ እወ እንተኮይኑ አንታይ ዓይነት መከላከሊ ጥንሲ ትጥቀሚ? | | | | 1. ከኒና  2. መርፍእ  3. ኣብ ኢድ ዝቅበር  4. ኣብ ማህን ዝቅመጥ  5 ካልእ እንተኮይኑ ጥቀሲ | | |  |
| 119 | ንሕቶ ቁፅሪ 118 መልስኪ ከኒና እንተኮይኑ ክንደይ ዓመት ተጠቂምኪ? | | | | ብዓመት-------------------- | | |  |
| ክፍሊ ክልተ፡ ሓበሬታ ብዛዕባ ናይ ኣፍ ደገ ማህፀን ካንሰርን ናይኣፍ ደገ ማህፀን ካንሰር ቅድመ ምርመራን ፍልጠት | | | | | | | |  |
| ተራ.ቁ | ሕቶታት | | መልስን & መማረፅታትን | | | | | skip |
| 200 | ብዛዕባ ናይ ኣፍ ደገ ማህፀን ካንሰር ሰሚዕኪ ትፈልጢ ዶ? | | 1.እወ  2. አይፈልጥን  መልስኪ አይፈልጥን እንተኮይኑ ናብ ሕቶ ቁፅሪ  210 ሕለፊ | | | | | If No Go to Q.401 |
| 201 | ንሕቶ ቁፅሪ 200 መልስኪ እወ እንተኮይኑ ካበይ ሰሚዕኪዮ? | | 1. ካብ መራኸብቲ ሓፋሽ ( ቴሌቭዥኝ፣ሬድዮ፣ ጋዜጣ)  2. ካብ በዓል ሙያ ጥዕና  3. ካብ ትምህርቲ  4. ካብ ቤተ ሰብ  5. ካብ መሓዛ  6. ካልእ እንተኮይኑ ጥቀሲ | | | | |  |
| 202 | ናይ ኣፍ ደገ ማህፀን ካንሰር ምልክታት እንታይ እዮም? (ካብ ሓደ ንላዕሊ ምምራፅ ይክአል እዩ) | | 1.ብብልዕቲ ደም ምፍሳስ  2. ብብልዕቲ ጨና ዘለዎ ፈሳሲ ምፍሳስ  3.ቃንዛ አብ እዋን ፆታዊ ርክብ  4. ድሕሪ ፆታዊ ርክብ ደም ምፍሳስ  5. አይፈልጥን  6.ካልእ እንተኮይኑ ጥቀሲ | | | | |  |
| 203 | ንኣፍ ደገ ማህፀን ካንሰር ዘቃልዑ ምክንያታት ትፈልጢ ዶ? | | 1. እወ  2.ኣይፈልጥን  መልስኪ አይፈልጥን እንተኮይኑ ናብ ሕቶ ቁፅሪ  205 ሕለፊ | | | | |  |
| 204 | ንሕቶ ቁፅሪ 203 መልስኪ እወ እንተኮይኑ ን ኣፍ ደገ ማህፀን ካንሰር ዘቃልዑ ምክንያታት እንታይ እዮም?  (ካብ ሓደ ንላዕሊ ምምራፅ ይክአል እዩ) | | 1. ዕድመ  2. ትሕቲ ዕደመ ፆታዊ ርክብ ምጅማር  3. ሕማም ብፆታዊ ርክብ ዝመሓላለፉ ሕማማት  4. ንነዊሕ ግዜ ብአፍ ዝውሰድ ናይ ጥንሲመከላኸሊ  ምጥቃም  5. ምሰ ብዙሓት ሰባት ፆታዊ ርክብ ምግባር  6. ኣብ ቤተ ሰብኩም ናይ ማህፀን ጫፍ ካንሰር ዘሎዎ  ነይሩኩም ዶ  7 ሲጋራ ምትካክ  8. አይፈልጥን  9. ካልእ እንተኮይኑ ጥቀሲ | | | | |  |
| 205 | ናይ ኣፍ ደገ ማህፀን ካንሰር ካብ ሰብ ናብ ሰብ ይመሓላለፍ ዶ? | | 1. እወ 2. ኣይመሓላለፍን 3. አይፈልጥን   መልስኪ ኣይመሓላለፍን ወይ አይፈልጥን  እንተኮይኑ ናብ ሕቶ ቁፅሪ 207 ሕለፊ | | | | | If NO go to Q.207 |
| 206 | ንሕቶ ቁፅሪ 205 መልስኪ እወ እንተኮይኑ ብከመይ ይመሓላለፍ? | | 1. ብፆታዊ ርክብ 2. ምስ ዝሓመመ ሰብ ብምንክካእ 3. ብአየር 4. አይፈልጥን 5. ካልእ እንተኮይኑ ጥቀሲ | | | | |  |
| 207 | ናይ ኣፍ ደገ ማህፀን ካንሰር ምክልከኻል ይክአል ዶ? | | 1.እወ  2.ኣይከአልን  3 አይፈልጥን  መልስኪ ኣይከአልን ወይ አይፈልጥን እንተኮይኑ  ናብ ሕቶ ቁፅሪ 209 ሕለፊ | | | | | If NO go to Q.210 |
| 208 | ንቁፅሪ 207 ሕቶ መልስኪ እወ እንተኮይኑ ብከመይ ምክልከኻል ይክአል? | | 1.ምስ ብዙሐት ሰባት ፆታዊ ርክብ ዘይምፍፃም  2.ትሕቲ ዕደመ ፆታዊ ርክብ ምውጋድ  3. ሽጋራ ዘይምትካኽ  4. ብክታበት  5. ናይ ቅድመ ካንሰር ምርመራ ብምግባር  6. አይፈልጥን  7. ካልእ እንተሃልዩ ጥቀሲ | | | | |  |
| 209 | ናይ ኣፍ ደገ ማህፀን ካንሰር ክድሕን ይኽእል ዶ? | | 1. እወ  2.ኣይድሕንን  3. ኣይፈልጥን | | | | | If NO go to  Q.212 |
| 210 | ብዛዕባ ኣፍ ደገ ማህፀን ካንሰር ቅድመ ምርመራ ሰሚዒኪ ትፈልጢ ዶ? | | 1. እወ  2. ኣይፈልጥን  መልስኪ አይፈልጥን እንተኮይኑ ናብ ሕቶ ቁፅሪ  301 ሕለፊ | | | | | If no skip to Q.301 |
| 211 | ንሕቶ ቁፅሪ 210 መልስኪ እወ እንተኮይኑ ካበይ ሰሚዕክዮ? | | 1. ካብ መራኸብቲ ሓፋሽ  2. ካብ በዓል ሙያ ጥዕና  3. ካብ ትምህርቲ  4 ካብ ቤተ ሰብ  5. ካብ መሓዛ  6. ካልእ እንተኮይኑ ጥቀሲ | | | | |  |
| 212 | ናይ ኣፍ ደገ ማህፀን ካንሰር ቅድመ ምርመራ ዕላሙ እንታይ እዩ? | | 1. ናይ ኣፍ ደገ ማህፀን ካንሰር ንምክልካል 2. ናይ ኣፍ ደገ ማህፀን ካንሰር ኣቀዲመካ ንምፍላይ 3. ናይ ኣፍ ደገ ማህፀን ካንሰር ንምሕካም 4. ካልእ እንተኮይኑ ጥቀሲ | | | | |  |
| 212 | ኣብ ደማ ኤችአይቪ ቫይረስ ዘለዋ ኣዶ ናይ ኣፍ ደገ ማህፀን ካንሰር ቅድመ ምርመራ መዓዝ ክትገብር ኣለዋ? | | 1. ወርሓዊ ፅግያት ምስ ጀመራ 2. ፆታዊ ርክብ ምስ ጀመረት 3. ዕድሚአ 30 ምስ መልኣት 4. ቆልዓ መውላድ ምስ ጀመረት 5. ናይ ኣዴታታ ምስገደፋ 6. ኣይፈልጥን 7. ካልእ እንተሃልዩ ጥቀሲ | | | | |  |
| 213 | \| ናይኣፍ ደገ ማህፀንካንሰርቅድመምርመራክንደይግዜክውሰድአለዎ? \| \| --- \| | | 1. ኣብ ዓመት ሓደ ግዜ  2 ኣብ ክልተ ዓመትሓደ ግዜ  3. ኣብ ሰለስተ ዓመትሓደ ግዜ  4. ኣብ ሓሙሽተ ዓመትሓደ ግዜ  5. ኣይፈልጥን  6. ካልእ እንተኮይኑ ጥቀሲ | | | | |  |
| 214 | ኣብ ከባቢኩም ናይ ኣፍ ደገ ማህፀን ካንሰር ቅድመ ምርመራ ዝህብ ጥዕና ትካል ትፈልጢ ዶ? | | 1.እወ  2. ኣይፈልጥን | | | | |  |
| ክፍሊ ሰለስተ፡ሓበሬታ ብዛዕባ ናይ ኣፍ ደገ ማህፀን ካንሰር ንናይ ኣፍ ደገ ማህፀን ካንሰር ቅድመ ምርመራን  ኣመለካክታ | | | | | | | | |
| 301 | ንናይ ኣፍ ደገ ማህፀን ካንሰር ናይ ምቅላዕ ዕድለይ ዝልዓለ እዩ ኢልኪ ዶ ትሓስቢ? | | | 1. እወ 2. ኣይኮኑኩን 3. አይፈልጥን | | | |  |
| 302 | ሓንቲ ኣዶ ብዉሑስ ዘይኮነ ፆታዊ ርክብ ናይ ኣፍ ደገ ማህፀን ካንሰር ክህልዋ ይክእል ዶ | | | 1. እወ 2. ኣይክእልን | | | |  |
| 303 | ናይ ኣፍ ደገ ማህፀን ካንሰር ሓደ ግዘ እንተተፈሊጡ ኣይድሕንን | | | 1. እወ 2. ይድሕን እዩ | | | |  |
| 304 | ኣብ ደመን ኤች አይ ቪ ቫይረስ ዘለወን ኣዴታት ብዝበለፀን ናይ ኣፍ ደገ ማህፀን ካንሰር ተቃላዕቲ እየን   \|  \| \| --- \| | | | 1. እወ 2. ኣይኮናን | | | |  |
| 305 | ሓንቲ ኣዶ ናይ ኣፍ ደገ ማህፀን ካንሰር እናሃለዋ ምልክት ዘይክተርኢትኽእል እያ ኢልኪ ትሓስቢ ዶ | | | 1 እወ  2 አይሓስብን  3 አይፈልጥን | | | |  |
| 306 | ናይ ኣፍ ደገ ማህፀን ካንሰር ቅድመ ምርመራ ብምግባር ናይ ኣፍ ደገማህፀን ካንሰር ምክልካል ይከኣል እዩ | | | 1. እወ 2. ኣይከኣልን | | | |  |
| 307 | ኩሉ ግዜ ኣፍ ደገ ማህፀን ካንሰር ቅድመ ምርመራ ንምግባር ንጥዕና በዓል ሞያ ንምምካር ፍቓደኛ ዲኪ | | | 1. እወ  2. ኣይኮኑኩን | | | |  |
| 308 | ናይ ኣፍ ደገማህፀን ካንሰር ቅድመ -ምርመራ ክገብር ዘለዎ መን እዩ ኢልኪ ትሓስቢ  (ካብ ሓደ ንላዕሊ ምምራፅ ይክአል እዩ) | | | 1.ኣብ ናይ ምውላድ ዕድመ ክልል ዘለዋ ኩለን  ኣዴታት  2.ናይማህፀን ጫፍ ካንሰር ዘርኢ ምልክት ጥራሕ  ዘለወን ኣዴታት  3. ኣብ ደመን ኤች አይ ቪ ቫይረስ ዘለወን ኣዴታት  4.ኣይፈልጥን | | | |  |
| 309 | አብ መጀመርታ ግዘ ፆታዊ ርክብ እንትትፍፅሚ ዕድምኪ ክንደይ ነይሩ | | | ብዓመት | | | |  |
| 311 | ምስ ክልተን ልዕሊኡን ሰባት ፆታዊ ርክብ ነይሩኪ ይፈልጥ ዶ | | | 1.እወ  2. ኣይነበረንን | | | |  |
| 312 | በዓል ገዛኺ ካሊእ ናይ ፆታዊ ርክብ መሓዛ ኣለቶ ዶ | | | 1. እወ 2. የብሉን 3. ኣይፈልጥን | | | |  |

የቅንየለይ
